# Essential Oils Composition and Biological Activity of *Chamaecyparis obtusa, Chrysopogon nigritanus* and *Lavandula coronopifolia* Grown Wild in Sudan

**DOI:** 10.3390/molecules28031005

**Published:** 2023-01-19

**Authors:** Loai M. H. Eltayeb, Sakina Yagi, Hanan M. M. Mohamed, Gokhan Zengin, Mohammad Ali Shariati, Maksim Rebezov, Abdullah Ibrahim Uba, Jose Manuel Lorenzo

**Affiliations:** 1 Department of Botany, Faculty of Science, University of Khartoum, Khartoum P.O. Box 321, Sudan; 2 Department of Biology, Science Faculty, Selcuk University, 42130 Konya, Turkey; 3 Department of Scientific Research, Russian State Agrarian University–Moscow Timiryazev Agricultural Academy, 127550 Moscow, Russia; 4 Department of Scientific Research, K. G. Razumovsky Moscow State University of Technologies and Management (The First Cossack University), 109004 Moscow, Russia; 5 Department of Scientific Research, V. M. Gorbatov Federal Research Center for Food Systems, 109316 Moscow, Russia; 6 College of Science and Mathematics, Rowan University, Glassboro, NJ 08028, USA; 7 Centro Tecnológico de la Carne de Galicia, Parque Tecnológico de Galicia, San Cibrao das Viñas, Avd. Galicia nº 4, 32900 Ourense, Spain; 8 Facultade de Ciencias, Universidade de Vigo, Área de Tecnoloxía dos Alimentos, 32004 Ourense, Spain

**Keywords:** antioxidant, *Chamaecyparis obtusa*, chemical profile, *Chrysopogon nigritanus*, enzyme inhibition, *Lavandula coronopifolia*

## Abstract

Generally, there are scant data about the constituents and eventually the biological activity of essential oils (EOs) from aromatic plants that grow naturally in Sudan. The present study aimed to determine the chemical composition, and antioxidant and enzyme inhibitory activities of EO extracted from the fruit of *Chamaecyparis obtusa* (Siebold and Zucc.) Endl. (family Cupressaceae), root of *Chrysopogon nigritanus* (Benth.) Veldkampis (family Poaceae) and aerial part of *Lavandula coronopifolia* Poir (family Lamiaceae). The fruit of *C. obtusa* contained only monoterpenes, mainly hydrogenated ones, with α-pinene (69.07%) as the major component. Oxygenated sesquiterpenes comprised the highest content of the *C. nigritanus* root EO with cedr-8-en-15-ol (28.69%) as the major constituent while aerial parts of *L. coronopifolia* contained both monoterpenes and sesquiterpenes and the oxygenated monoterpene lavandulol (26.56%) as dominant compounds. The EO of the root of *C. nigritanus* significantly displayed (*p* < 0.05) the highest anti-DPPH radical, Fe^3+^- and Cu^2+^-reducing and metal-chelating activities, while that of *C. obtusa* fruit significantly exerted (*p* < 0.05) the best anti-ABTS radical and total antioxidant activity. The two EOs significantly exhibited (*p* < 0.05) the highest anti-acetylcholinesterase and -butyrylcholinesterase activities, respectively, while EO of *L. coronopifolia* was the only oil to show a considerable inhibitory effect against the tyrosinase and α-glucosidase enzymes. In conclusion, EOs from these three plants could be natural agents with promising functional properties for food, cosmetics, and pharmaceutical applications.

## 1. Introduction

Essential oils (EOs) extracted from various herbs and spices have been a subject of intensive research, partially due to the continuous discoveries of their multifunctional properties beside their classical roles as food additives, preservatives and/or fragrances [1]. More than 3000 plants are known to produce Eos, of which approximately 300 are of commercial importance [2]. They are obtained from all parts of the plant and contain a high variety of volatile, aromatic and low-molecular-weight compounds. They are generally extracted by conventional methods like hydrodistillation and steam distillation. However, more advanced alternative microwave-based techniques like microwave-assisted hydrodistillation, microwave steam distillation and solvent-free microwave extraction have been proven to be fast, secure and efficient [3,4]. The EO’s market size was $9.62 billion in 2021 and is expected to reach $18.25 billion in 2028 [5]. The greatest countries to produce EOs include India, China, Brazil, France and Indonesia, while Africa, Morocco, Tunisia, Egypt, and Algeria are the largest exporters of EOs. The greatest consumers of EOs are the United States (40%), European Union (30%) and Japan (7%) [6]. 

In Sudan, EOs extracted from aromatic plants have a rich history of use as a source of food additive and preservative in addition to their medicinal and cosmetic applications. For example, the species *Chamaecyparis obtusa* (Siebold and Zucc.) Endl. (from the Cupressaceae family), locally known as Shagarat Bakhor Alanfar, is an aromatic plant grown in Western Sudan. EOs are mainly extracted from leaves, fruits, and wood. The plant is used in Sudan to treat abdominal disease [7]. EOs from this plant were found to possess antibacterial, antifungal [8,9], larvicidal [10] and insecticidal [11,12] activities and suppress the activity of house dust mites in tatami mats [13,14]. Additionally, EOs were found to be effective for the treatment of atopic dermatitis [15], respiratory inflammation-related disease [16] and to promote hair growth in shaved mice [17]. The fruit was found to be rich in alkaloids, flavonoids, sterols, tannins, triterpenes, cardiac glycosides and phenols [7]. Seven new compounds, including one dimer novel skeleton, chamaecyformosanin A, in addition to six terpenes were isolated from the methanol extract of the bark of *C. obtusa* var. *formosana* and three metabolites presented antibacterial activities [18].

Another example is *Chrysopogon nigritanus* (Benth.) Veldkampis (Syn: *Vetiveria nigritana* (Benth.) Stapf) (family Poaceae), known locally as Rwag almouya, which is a perennial grass grown mainly in Western Sudan. The roots are sold in the market as a water purifier and they give the drinking water a “better scent”. Macerations or infusions of roots were used as an antidiarrheal for children [19]. The sesquiterpenes α-vetivone, β-vetivone and khusimol are considered the “fingerprint” of vetiver oil. Carlinoside, neocarlinoside, 6, 8-di-*C*-arabinopyranosylluteolin, isoorientin and tricin-5-*O*-glucoside were isolated from the root ethanolic extract of a plant grown in Mali [20]. 

*Lavandula coronopifolia* Poir. (family Lamiaceae), known locally in Sudan as Sedam, is a fragrant perennial herb that grows naturally in mountainous slopes of Red Hills in Eastern Sudan and in Jebbel Marra and Jebel Meridob in Western Sudan [21]. The powder of the whole plant is applied on wounds to stop bleeding [19]. Carvacrol (48.9%), (*E*)-caryophyllene (10.8%) and caryophyllene oxide (7.7%) were the dominant constituents of the EO of *L. coronopifolia* grown in Morocco [22]. Aerial parts of the plants have shown to possess larvicidal, antibacterial, antibiofilm [23], antioxidant [24], antimicrobial [25], α-glucosidase inhibitory [26], and hepatoprotective [27] activities. Hydroxyl flavones like hypolaetin, isoscutellarien, and luteolin [28] as well as polyhydroxyoleanolic acids, polyhydroxyursolic acids and their glycosides, caffeic acid, rosmarinic acid, rutin, quercetin, and hesperidin [26,29] have been identified from the aerial parts.

Understanding the EOs’ composition of herbs is necessary to predict its specific mode of action, and therefore the possible therapeutic outcome [30]. Although many plant species from Sudan have been described as a rich source of essential oils with potential biological activities, the number of high-impact scientific studies in this area is relatively low. In fact, there are scant data about EOs from the above-mentioned aromatic plants that grow naturally in Sudan, and also no or few data on their antioxidant and enzyme inhibition activities have been reported. Therefore, in continuation of our work on aromatic plants indigenous to Sudan, the present study has been set up to determine the chemical profile of EOs extracted from the fruit of *C. obtusa*, root of *C. nigritanus* and aerial parts of *L. coronopifolia* and evaluate their antioxidant and enzyme inhibition activities with the aim of gaining insights into their application potential and seeking alternatives to synthetic chemicals that present risks to human health and to the environment. Antioxidant activity tests included scavenging of free radicals, reduction potential, phosphomolybdenum, and chelating ability. The enzyme inhibitory property of the essential oils was evaluated on enzymes related with neurodegenerative ailments (acetylcholinesterase (ACh) and butyrylcholinesterase (BCh)), diabetes and obesity (α-glucosidase and α-amylase) and skin hyperpigmentation tyrosinase (Tyr) enzymes.

## 2. Results and Discussion

### 2.1. Chemical Profiles of the EOs

**Figure 1:**
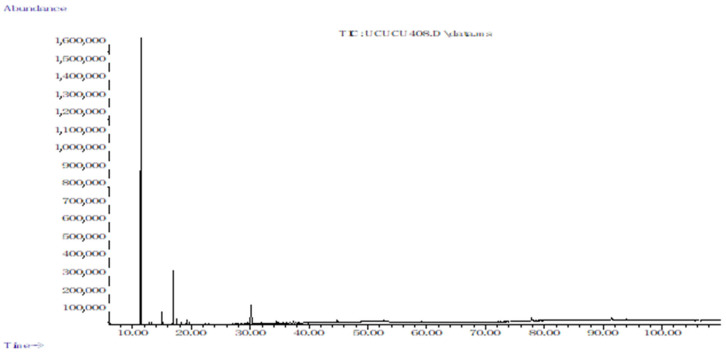
Total ion chromatogram of essential oil from fruit of *Chamaecyparis obtusa*.

**Figure 2:**
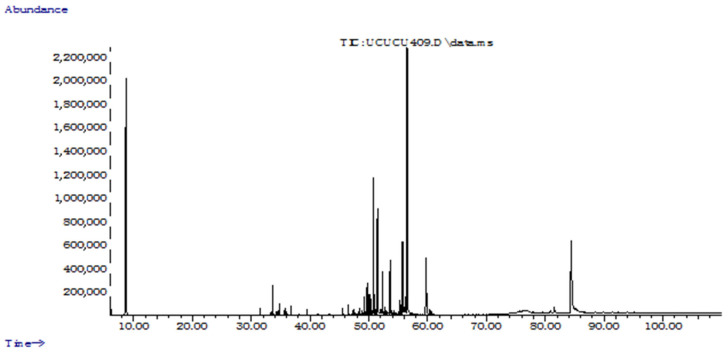
Total ion chromatogram of essential oil from root of *Chrysopogon nigritanus*.

**Figure 3:**
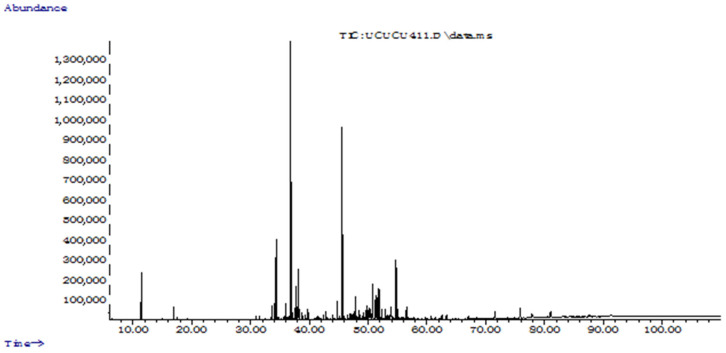
Total ion chromatogram of essential oil from aerial part of *Lavandula stricta*.

Hydrodistillation of dried fruit of *C. obtusa* gave a yellow-coloured EO with a sweet odour and a yield of 0.8% (*w*/*w*). Results of chemical profiles are depicted in Table 1. Chromatograms are presented in Figure 1, Figure 2 and Figure 3. The total number of eight compounds belonging to monoterpenes was identified. Hydrogenated and oxygenated monoterpenes represented 87.3% and 11.6% of the total composition of EO. α-Pinene (69.1%) was the dominant compound, followed by δ-3-carene (12.1%) and *trans*-sabinene hydrate (11.6%), respectively. Sesquiterpenes were not detected. It worth mentioning that this is the first report on EO composition of the fruit. Previous studies on *C. obtusa* EO were only performed on the leaf of trees grown in South Korea, where it was found to also be rich in monoterpenes [9,16,31].

Extraction of EO from *C. nigritanus* root yielded 1.1% (*w*/*w*) of a yellow-coloured oil with an aromatic-spicy odor. Chemical analysis revealed a total of 19 compounds (Table 2). The majority of compounds were oxygenated sesquiterpenes (64.3%) with Cedr-8-en-15-ol (28.7%) as the major constituent. Sesquiterpene hydrocarbons represented 4.0% of the EO while monoterpenes were not detected. Moreover, the alkane triacontane (20.1%) was found in a relatively abundant amount. These results were contradictory to those obtained by Khalil and Ayoub [32] and Semde et al. [33] but generally in agreement with that performed by Champagnat et al. [20]. The latter found that the EO from the root of a plant grown in Mali contained only sesquiterpene terpenes among them the alcohols preziza-7(15)-en-12-ol (9.5%), cedren-8-en-15-ol (6.2%), preziza-7(15)-en-3α-ol (6.0%), preziza-7(15)-ene (1.7%) and β-cedrene (1.6%). In the present study, Cedr-8-en-15-ol (28.7%) and α-Cedrene (2.0%) were the major oxygenated and hydrocarbon sesquiterpenes, respectively, and preziza-7(15)-ene was not detected. Khalil and Ayoub [32] and Semde et al. [33] reported that the sesquiterpene hydrocarbons longifolene-D (25.2%) and dehydronigritene (25.1%) were the main components of the EO root of *C. nigritanus* grown in Sudan and Burkina Faso, respectively. Additionally, α- and β-vetivone and khusimol, which are considered the “fingerprint” of vetiver oil, were not detected in the present study, thus suggesting the presence of a different chemotype of *C. nigritanus* characterized by the presence of high amounts of Cedr-8-en-15-ol (28.7%). Evidently, this aspect needs additional confirmations with further studies. On the other hand, this variation could also be attributed to maturity of the plant and environmental factors. In fact, studies have shown that the terpenoid composition of aromatic plants can be affected by the photoperiod, light intensity, temperature, rainfall and harvesting season or month [34,35]. De Almeida et al. [36] reported that multiple environmental factors change the specialized metabolism on plants, resulting in production of different terpenes.

Aerial parts of *L. coronopifolia* yielded 0.2% (*w*/*w*) of a nicely perfumed faint yellow-coloured EO. The chemical profile of the EO is presented in Table 3. Nineteen compounds were identified and sesquiterpenes comprising 58.6% of the EO with the majority belonged to oxygenated sesquiterpenes (41.5%), while monoterpenes forming 32.9% of the EO with only one oxygenated monoterpenes lavandulol (26.6%) was detected and represented the main constituent of the EO. This was followed respectively by caryophyllene oxide (18.4%), farnesyl acetone (8.0%) and β-gurjunene (7.4%). Only one previous study by Alizadeh and Aghaee [37] reported the composition of EO of *L. coronopifolia.* They found that the EO of the aerial part of a plant grown in the Genow and Rodan regions in Iran was mainly composed of hydrocarbon monoterpenes (73.7–75.1%) and oxygenated monoterpenes (24.6–25.8%) with α-pinene (58.3–63.5%) as the main component. In this study, although lavandulol (26.6%), an oxygenated monoterpenes, was the major compound, the EO was mainly dominated by oxygenated sesquiterpenes compounds and only two hydrogenated monoterpenes; namely, α-pinene (5.0%) and δ-3-carene (1.3%) were detected. The prevalence of sesquiterpenes could be associated to the harsh environmental conditions of the plant, as a previous study showed that sesquiterpenes are effective in the defense against biotic and abiotic stresses, thereby protecting the plants [38]. The characteristic nice fragrance of the EO can be due to the dominance of lavandulol, which is known to play a significant role in the perfume and cosmetics industry [39].

### 2.2. Antioxidant Activity

Antioxidants have been widely used as food additives to provide protection against oxidative degradation of foods by free radicals, and they also have many beneficial effects for human health. Six complementary assays were performed to evaluate the antioxidant activity of the three essential oils under investigation. Results are presented in Table 4. The antiradical activity was evaluated by testing the capacity of the EOs to scavenge the free DPPH and ABTS radicals and results showed that EO extracted from the root of *C. nigritanus* displayed significantly (*p* < 0.05) higher anti-DPPH activity than that extracted from the aerial parts of *L. coronopifolia,* while EO from *C. obtusa* was not active. On the other hand, the EO from the latter significantly exerted (*p* < 0.05) the highest anti-ABTS activity, followed by those obtained from the *L. coronopifolia* aerial part and *C. nigritanus* fruit, respectively. Furthermore, EO from *C. nigritanus* root significantly exhibited (*p* < 0.05) the highest Cu^++^ and Fe^+++^ ion-reducing capacity, followed by EO from the *C. obtusa* fruit and *L. coronopifolia* aerial parts, respectively. EOs from both *C. obtusa* fruit and *C. nigritanus* root were not significantly (*p* ≥ 0.05) different in their metal-chelating capacity, while that of *L. coronopifolia* aerial parts revealed lower activity. The total antioxidant activity from the phosphomolybdenum assay showed that the EO of *C. obtusa* fruit was significantly (*p* < 0.05) 2.6- and 7.1-fold highly active than those recorded from the *C. nigritanus* root and *L. coronopifolia* aerial parts, respectively. Overall, the high antioxidant activity of *C. nigritanus* root in most assays could have been due to the richness of the EO with oxygenated sesquiterpenes (64.3%) [40], among them α-bisabolol which was reported to ameliorate the antioxidant network and re-establish the redox balance by antagonising oxidative stress [41]. Results of *L. coronopifolia* aerial parts supported those obtained by Messaoud et al. [24] who studied the anti-DPPH (IC_50_ 162.2 µgmL^−1^), Ferric-reducing (60.7 µmol g^−1^) and metal-chelating (IC_50_ 6.1 µgmL^−1^) activities of a species grown in Tunisia. They related this antioxidant activity to the richness of EO in hydrocarbon monoterpenes and/or to the synergistic effect of more than one individual oil compound as well as compounds like carvacrol, thymol methyl ether, and carvacrol methyl ether [42]. Furthermore, α-pinene might partly contribute to the observed antioxidant activity of EO of *C. obtusa* fruit [43].

### 2.3. Enzyme-Inhibitory Activity

The capacity of the three EOs under investigation to inhibit the AChE, BChE, Tyr, α-glucosidase and α-amylase enzymes was also examined and results are presented in Table 5. The EO of *C. obtusa* fruit significantly exerted (*p* < 0.05) the highest AChE inhibition activity followed by that of *L. coronopifolia* aerial parts. This high activity could be attributed to α-pinene and δ-3-carene, the major components of *C. obtusa* fruit EO, which was known for their potential as anti-AChE [44,45]. Although the EO of the *C. nigritanus* root did not show any anti-AChE activity, it significantly exerted (*p* < 0.05) the highest BChE inhibition activity followed by EO of *C. obtusa* fruit, while that from *L. coronopifolia* aerial parts was not active. Only, EO of the latter species displayed considerable anti-Tyr activity. However, water extracts of dried lavender leaves from *L. angustifolia, L. stoechas, L. dentata* and *L. latifolia* grown in northern Iran have been found to possess anti-Tyr activity suggesting that *Lavandula* spp. could be an interesting source of tyrosine inhibitor agents [46]. Results of the inhibition property against the two enzymes associated with diabetes and obesity showed that EOs of both *C. obtusa* fruit and *L. coronopifolia* aerial part had the same capacity to inhibit the α-amylase with a value significantly (*p* < 0.05) higher than that exerted by the EO of *C. nigritanus* root. However, only EO from *L. coronopifolia* aerial parts exerted inhibition activity against α-glucosidase enzyme in concurrence with the findings of Elsbaey et al. [26]. Moreover, Boutahiri et al. [47] reported that the aqueous crude extract of flower tops of *L. pedunculate* inhibited the pancreatic α-amylase and intestinal α-glucosidase enzyme activities, suggesting that this genus may be a good source of antidiabetic agents.

### 2.4. Molecular Docking

**Figure 4:**
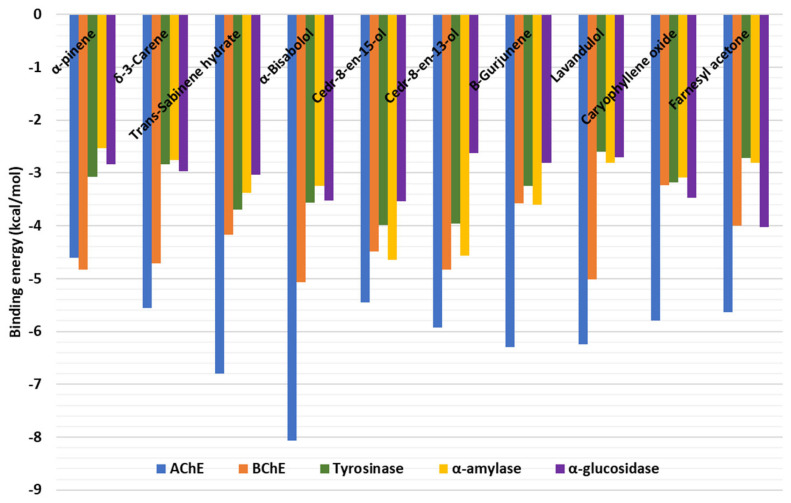
Binding energy (docking) scores of phytochemicals extracted from *Chamaecyparis obtusa, Chrysopogon nigritanus* and *Lavandula coronopifolia*.

**Figure 5:**
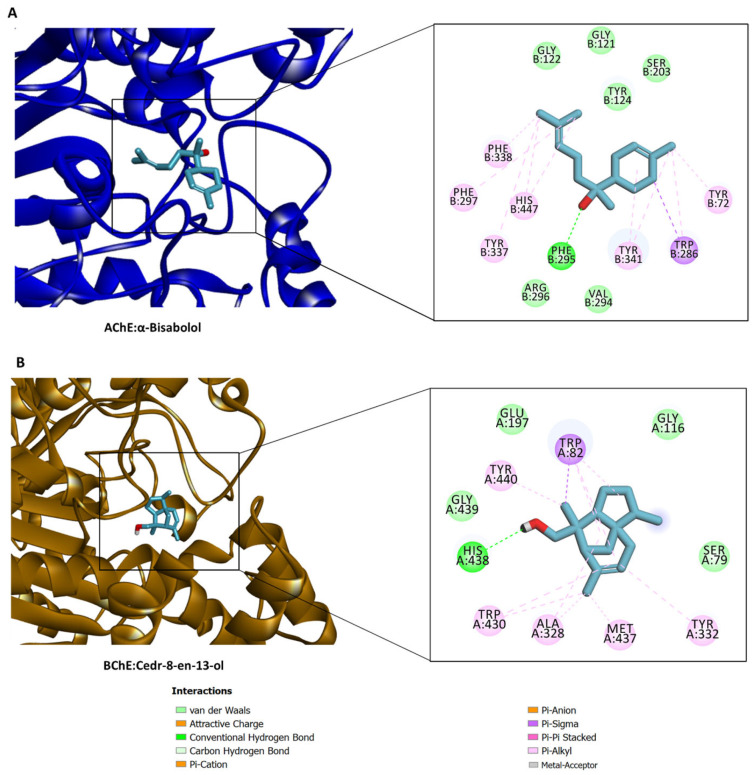
Protein–ligand interaction: (**A**) AChE and α-bisabolol and (**B**) BChE and cedr-8-en-13-ol. These phytochemical compounds were extracted from *C. obtusa, C. nigritanus* and *L. coronopifolia*.

**Figure 6:**
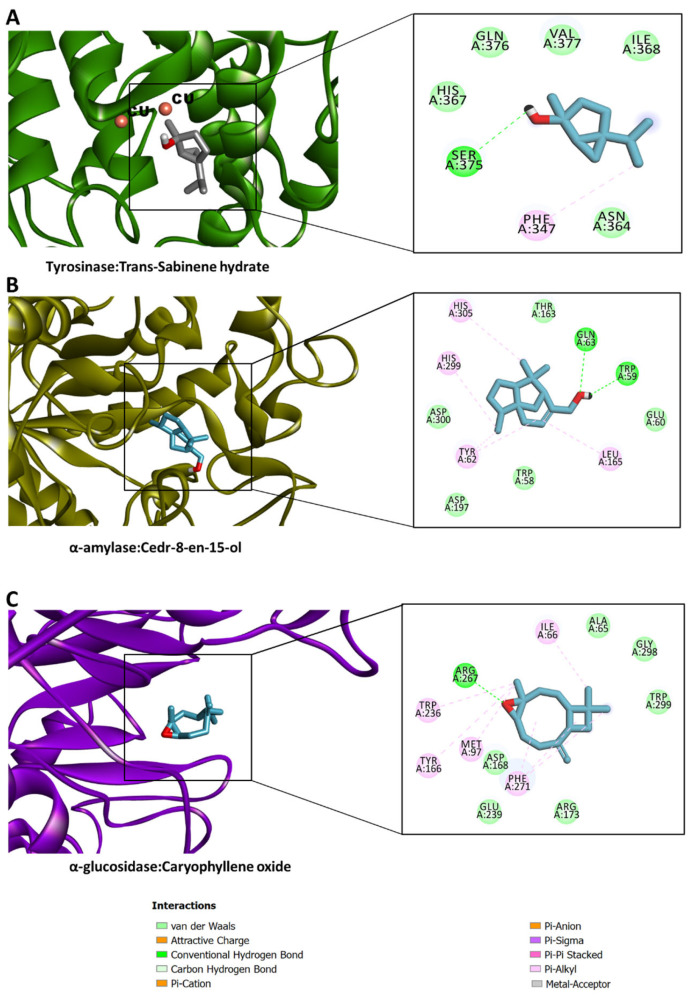
Protein–ligand interaction: (**A**) tyrosinase and *trans*-sabinene hydrate, (**B**) α-amylase and cedr-8-en-15-ol, and (**C**) α-glucosidase and caryophyllene oxide. These phytochemical compounds were extracted from *C. obtusa, C. nigritanus* and *L. coronopifolia*.

All docked compounds bound to all five enzymes, with a potential preference for AChE and BChE (Figure 4). The detailed protein–ligand interactions provided insights into the binding patterns of some selected compounds. All the study compounds were quite small molecules, hence freely bound to the active sites of the target proteins. α-Bisabolol was accommodated in the catalytic channel of AChE via an H-bond with Phe295 and multiple hydrophobic interactions, while van der Waals interactions reinforced the binding (Figure 5A). Similarly, cedr-8-en-13-ol occupied the catalytic pocket of BChE, mainly via an H-bond with His438, and multiple hydrophobic interactions, with a couple of van der Waals interactions contributing to the overall binding affinity (Figure 5B). Interestingly, *trans*-sabinene hydrate fit in the relatively narrow catalytic channel of tyrosinase, forming an H-bond via a hydroxyl group with Ser375, a hydrophobic interaction, and a couple of van der Waals interactions; however, the active-site copper metal ions were not engaged in any interactions (Figure 6A). In the case of α-amylase, cedr-8-en-15-ol formed two H-bonds with Trp59 and Gln63 via a hydroxyl group, a couple of hydrophobic interactions, and van der Waals interactions with the active site residues (Figure 6B). Additionally, caryophyllene oxide is completely buried in the catalytic cavity of α-glucosidase, forming an H-bond with Arg267, multiple hydrophobic and van der Waals interactions all over the channel (Figure 6C). Taken together, H-bonds and hydrophobic contacts are the key interactions with which the selected phytochemicals from *C. obtusa, C. nigritanus,* and *L. coronopifolia* extracts bind to, and potentially inhibit these enzymes.

## 3. Materials and Methods

### 3.1. Plant Materials

Plant samples were collected from their natural habitat in Sudan. Fruits of *C. obtusa* and roots of *C. nigritanus* were collected in February 2022, from El Obeid, North Kordofan, and Western Sudan (Latitude: 13°11′3″ N. Longitude: 30°13′0″ E.), while aerial parts (stems and leaves) of *L. coronopifolia* were harvested in December 2021 from Erkowit, Eastern Sudan (Latitude: 18°42′0″ N. Longitude: 37°0′0″ E). Plants were taxonomically identified by Prof. Maha Kordofani, and voucher specimens (No. 2023/2CO for *C. obtusa*, No. 2023/2CN for *C. nigritanus* and No. 2022/11LC for *L. coronopifolia*) were deposited in the Herbarium of the Botany Department, Faculty of Science, University of Khartoum, Sudan.

### 3.2. Extraction of Essential Oils

EO from each plant sample (400 g) was extracted by hydrodistillation using a Clevenger-type apparatus for 4 h. The extracted oils were dried over anhydrous sodium sulphate and stored at 4 °C, in amber-coloured bottles, before use.

### 3.3. Chemical Analysis of the EOs

The essential oil composition was characterized by gas chromatography-flame ionization detector (GC-FID) and gas chromatography-mass spectrophotometry (GC-MS) techniques. GC-MS analysis was performed by using an Agilent 5975 GC-MS system coupled to an Agilent 7890 A GC. To separate chemical components, the HP-Innowax column (60 m × 0.25 mm, 0.25 μm film thickness) was used. Other analytical parameters were reported in our earlier paper [48].

The retention index (RI) calculated by co-injection with reference to a homologous series of *n*-alkanes (C8–C30) under the identical experimental circumstances was used to identify the components. In order to make more accurate identifications, RI values and mass spectra of the compounds were compared with those of the literature and NIST 05 and Wiley eighth edition, respectively.

### 3.4. Antioxidant and Enzyme Inhibitory Assays

The antioxidant and enzyme inhibitory activity of comfrey root extracts was determined according to previously described methods [49,50]. DPPH and ABTS radical scavenging activity, cupric ion-reducing antioxidant capacity (CUPRAC), and ferric ion-reducing antioxidant power (FRAP) were expressed as mg trolox equivalents (TE)/g oil. The metal chelating ability (MCA) was reported as mg EDTA equivalents (EDTAE)/g oil, whereas the total antioxidant activity (phosphomolybdenum assay, PBD) was expressed as mmol TE/g extract. AChE and BChE inhibitory activities were given as mg galanthamine equivalents (GALAE)/g oil; tyrosinase inhibitory activity was expressed as mg kojic acid equivalents (KAE)/g extract; amylase and glucosidase inhibitory activities were presented as mmol acarbose equivalents (ACAE)/g oil.

### 3.5. Molecular Modeling

The crystal structures of target enzymes: AChE (PDB ID: 6O52) [51], BChE (PDB ID: 6EQP) [52], and α-amylase (PDB ID: 1B2Y) [53] were retrieved from the protein data bank (PDB) (https://www.rcsb.org/) (accessed on 6 October 2022). However, since the crystal structures of human α-tyrosinase and α-glucosidase are not available, those of *Priestia megaterium* tyrosinase (6QXD) [54] and *Mus musculus* α-glucosidase (7KBJ) [55] were used as templates to build their human models using their respective UniProt sequences P14679, and P0DUB6. The details of the homology modeling are available in the Ref. [56]. All protein structures were prepared according to the protocol described in the Ref. [57]. The 3D structures of selected ligands were downloaded from the PubChem database (https://pubchem.ncbi.nlm.nih.gov/) (accessed on 23 December 2022) and geometry optimization was done using Biovia Discovery Studio Visualizer v4.5 (Dassault Systèmes Biovia Software Inc., San Diego, CA, USA, 2012). The cocrystal ligand in each complex was used to define the docking grid box dimension and coordinates using AutoDockTools 1.5.6, and each ligand was docked into the binding pocket of each protein using AutoDock 4.2.6 (https://autodock.scripts.edu accessed on 15 December 2022) [58]. The details of the docking protocol have been described in the Ref. [59]. The docking score of each ligand against each protein was estimated in terms of binding energy, and protein–ligand interactions were visualized using Biovia Discovery Studio Visualizer v4.5 (Dassault Systèmes Biovia Software Inc., San Diego, CA, USA, 2012).

### 3.6. Statistical Analysis

Data are presented as mean ± standard deviation of the number (*n* = 3) of replicates. One-way analysis of variance with Tukey’s post hoc test was conducted; *p* < 0.05 was considered statistically significant. The statistical evaluation was performed using Graphpad version 9.0.

## 4. Conclusions

Chemical analysis of the three EOs indicated that the fruit of *C. obtusa* contained only monoterpenes, mainly hydrogenated ones, with α-pinene as the major component. The *C. nigritanus* root composed only of sesquiterpenes with a majority of compounds are oxygenated ones and Cedr-8-en-15-ol as a dominant component, while aerial parts of *L. coronopifolia* contained both monoterpenes and sesquiterpenes, but with higher content in the latter and lavandulol as the major compound. It was clear that the three investigated EOs were varied in their antioxidant and enzyme inhibition properties, and this is possible due to the variation in their chemical composition. Generally, the EO of the *C. nigritanus* root displayed the best antioxidant activity (4/6 assays). The EO of the *L. coronopifolia* aerial part was the only EO to reveal anti-Tyr and–α-glucosidase activities. Additionally, it displayed, like that of the *C. obtusa* fruit, the best α-amylase inhibition capacity. EOs of the *C. obtusa* fruit and *C. nigritanus* root revealed the best anti-AChE and -BChE activities, respectively. Molecular docking was performed to get insights into the binding mode and interaction of the bioactive compounds with the studied enzyme targets. The results showed that the docked compounds bound to all five enzymes, with a potential preference for AChE and BChE by α-Bisabolol and cedr-8-en-13-ol, respectively. Together, these data show that EOs could have significant potential in different food, cosmetic and pharmaceutical applications. Further, studies are recommended to evaluate their biosafety and determine whether these biological activities are attributed to their main components of the EOs or the synergistic effect of more than one compounds. Additionally, understanding the genetic factors and environmental conditions that alter the composition and quantity of volatile compounds is essential for pharmacological studies.

## Figures and Tables

**Table 1:** Chemical composition of essential oils from fruit of *Chamaecyparis obtusa*.

No.	RIL ^a^	RI ^b^	Compounds	Percentage Occurrence (%)
1	1032	1023	α-Pinene	69.1
2	1065	1057	α-Fenchene	0.7
3	1076	1068	Camphene	0.4
4	1118	1111	β-pinene	3.1
5	1159	1157	δ-3-Carene	12.1
6	1173	1174	Myrcene	1.1
7	1203	1201	Limonene	0.8
8	1474	1473	*Trans*-Sabinene hydrate	11.6
	Total hydrogenated monoterpenes			87.3
	Total oxygenated monoterpenes			11.6
	Total identified			98.9

^a^ Relative retention indices for each compound in the literature. ^b^ Relative retention indices calculated against *n*-alkanes. The compounds have been sorted according to their retention indices on an HP-Innowax polar column.

**Table 2:** Chemical composition of essential oil from root of *Chrysopogon nigritanus*.

No.	RIL ^a^	RI ^b^	Compounds	Percentage Occurrence (%)
1	1577	1576	α-Cedrene	2.0
2	1610	1601	β-Gurjunene	0.1
3	1612	1616	β-Caryophyllene	0.7
4	1651	1652	Sabina ketone	0.4
5	1688	1689	Selina-4,11-diene	0.5
6	1786	1779	Ar-Curcumene	0.3
7	2008	2010	Caryophyllene oxide	0.4
8	2050	2050	(*E*)-Nerolidol	0.7
9	2088	2084	Cubenol	0.4
10	2170	2162	β-Bisabolol	1.7
11	2182	2184	α-Cedrol	2.2
12	2187	2192	T-Cadinol	1.7
13	2232	2231	α-Bisabolol	10.0
13	2250	2257	β-Eudesmol	8.3
15	2291	2293	1,4-Dimethyl azulene	3.7
16	2348	2340	Widdrol	4.9
17	2411	2413	4-Iso propil-6-methyl-1-tetra-1-one	7.0
18	2540	2436	Cedr-8-en-15-ol	28.7
19	2530	2528	Cedr-8-en-13-ol	5.3
20	3000	3000	Triacontane	20.1
	Total sesquiterpene hydrocarbons			4.0
	Total oxygenated sesquiterpenes			64.3
	Others			30.8
	Total identified			99.1

^a^ Relative retention indices for each compound in the literature. ^b^ Relative retention indices calculated against *n*-alkanes. The compounds have been sorted according to their retention indices on an HP-Innowax polar column.

**Table 3:** Chemical composition of essential oil from the aerial part of *Lavandula stricta*.

No.	RIL ^a^	RI ^b^	Compounds	Percentage Occurrence (%)
1	1032	1023	α-Pinene	5.0
2	1159	1157	δ-3-Carene	1.3
3	1577	1576	α-Cedrene	1.3
4	1600	1600	β-Elemene	1.6
5	1610	1601	β-Gurjunene	7.4
6	1651	1652	Sabina ketone	2.6
7	1686	1685	Lavandulol	26.6
8	1708	1713	Ledene ((+)-Viridiflorene)	3.5
9	1726	1727	Germacrene	1.7
10	1727	1730	7-Epi-1,2-dehydrosesquicineole	4.8
11	1773	1778	γ-Cadinene	1.6
12	2008	2010	Caryophyllene oxide	18.4
13	2104	2100	Viridiflorol	1.9
13	2187	2192	T-Cadinol	1.4
15	2219	2208	T-Muurolol	1.1
16	2232	2231	α-Bisabolol	3.6
17	2255	2250	α-Cadinol	2.4
18	2298	2273	Capric acid	3.2
19	2384	2381	Farnesyl acetone	8.0
	Total hydrogenated monoterpenes			6.3
	Total oxygenated monoterpenes			26.6
	Total sesquiterpene hydrocarbons			17.1
	Total oxygenated sesquiterpenes			41.5
	Others			5.8
	Total identified			97.3

^a^ Relative retention indices for each compound in the literature. ^b^ Relative retention indices calculated against *n*-alkanes. The compounds have been sorted according to their retention indices on an HP-Innowax polar column.

**Table 4:** Antioxidant activity of the essential oil of the investigated aromatic plants.

Plant Name	DPPH (mg TE */g)	ABTS (mg TE/g)	CUPRAC (mg TE/g)	FRAP (mg TE/g)	MCA (mg EDTAE **/g)	PBD (mmol TE/g)
*C. obtusa* fruit	na	20.6 ± 0.9 ^a^	125.1 ± 3.2 ^b^	71.0 ± 0.6 ^b^	37.7 ± 1.9 ^a^	51.5 ± 0.1 ^a^
*C. nigritanus* root	1.4 ± 0.3 ^a^	12.8 ± 0.9 ^c^	188.4 ± 0.7 ^a^	120.1 ± 0.3 ^a^	39.2 ± 2.9 ^a^	19.5 ± 0.0 ^b^
*L. coronopifolia* aerial part	0.6 ± 0.1 ^b^	17.5 ± 1.0 ^b^	73.5 ± 1.9 ^c^	30.2 ± 0.5 ^c^	33.0 ± 0.7 ^b^	7.2 ± 0.2 ^c^

Values are reported as mean ± SD. DPPH: 2,2-diphenyl-1-picrylhydrazyl, ABTS: 2,2’-azino-bis(3-ethylbenzothiazoline-6-sulfonic acid), CUPRAC: cupric ion reducing antioxidant capacity, FRAP: ferric reducing antioxidant power, MCA: metal chelating activity, PBD: phosphomolybdenum. * TEs, trolox equivalents; ** EDTAEs, disodium edetate equivalents; Different superscript letters in the same column indicate significant difference (*p* < 0.05).

**Table 5:** Enzyme inhibitory activity of the essential oil of the investigated aromatic plants.

Plant Name	AChE (mg GALAE/g) *	BChE (mg GALAE/g)	Tyrosinase (mg KAE/g) **	Amylase (mmol ACAE/g)	Glucosidase (mmol ACAE/g) ***
*C. obtusa* fruit	2.5 ± 0.1 ^a^	2.2 ± 0.2 ^b^	na	0.5 ± 0.0 ^a^	na
*C. nigritanus* root	na	3.3 ± 0.0 ^a^	na	0.5 ± 0.0 ^b^	na
*L. coronopifolia* aerial part	1.8 ± 0.1 ^b^	na	34.3 ± 0.7	0.5 ± 0.0 ^a^	1.4 ± 0.0

Values are reported as mean ±SD; * GALAEs, galanthamine equivalents; ** KAEs, kojic acid equivalents; *** ACEs, acarbose equivalents; Different superscript letters in the same column indicate significant difference (*p* < 0.05); na, not active.

## Data Availability

Not applicable.

## References

[1] Sharifi-Rad J., Sureda A., Tenore G.C., Daglia M., Sharifi-Rad M., Valussi M., Tundis R., Sharifi-Rad M., Loizzo M.R., Ademiluyi A.O. (2017). Biological Activities of Essential Oils: From Plant Chemoecology to Traditional Healing Systems. Molecules.

[2] Burger P., Plainfossé H., Brochet X., Chemat F., Fernandez X. (2019). Extraction of natural fragrance ingredients: History overview and future trends. Chem. Biodivers..

[3] Mohammadhosseini M. (2015). Chemical composition of the essential oils and volatile fractions from flowers, stems and roots of *Salvia multicaulis* Vahl. by Using MAHD, SFME and HS-SPME Methods. J. Essent. Oil Bear. Plants.

[4] Mohammadhosseini M., Venditti A., Mahdavi B. (2023). Characterization of essential oils and volatiles from the aerial parts of *Mentha pulegium* L. (Lamiaceae) using microwave-assisted hydrodistillation (MAHD) and headspace solid phase microextraction (HS-SPME) in combination with GC-MS. Nat. Prod. Res..

[5] (2021). Market Research Report, Essential Oils Market Size, Share & Growth Report [2021–2028]. https://www.fortunebusinessinsights.com.

[6] Marques M.O.M., Facanali R., Haber L.L., Vieira M.A.R. (2012). Essential oils: History, biosynthesis, and agronomic aspects. Med. Essent. Oils Chem. Pharmacol. Ther. Asp..

[7] Elshiekh Y.H., Ali M.A.M., Abubakr R., Musa Y.A.A. (2020). Evaluation of Antibacterial, Antioxidant and Phytochemical screening of *Chamaecyparis obtusa* (Crippsii) Fruits. Am. J. Res. Commun..

[8] Koyama S., Yamaguchi Y., Tanaka S., Motoyoshiya J. (1997). A new substance (Yoshixol) with an interesting antibiotic mechanism from wood oil of Japanese traditional tree (Kiso-Hinoki), *Chamaecyparis obtusa*. Gen. Pharmacol..

[9] Yang J.-K., Choi M.-S., Seo W.-T., Rinker D.L., Han S.W., Cheong G.-W. (2007). Chemical composition and antimicrobial activity of *Chamaecyparis obtusa* leaf essential oil. Fitoterapia.

[10] Jeon J.-H., Lee S.-H., Kim M.-K., Lee H.-S. (2005). Larvicidal activity of *Chamaecyparis obtusa* and *Thuja orientalis* leaf oils against two mosquito species. J. Appl. Biol. Chem..

[11] Lee S.-H., Do H.-S., Min K.-J. (2015). Effects of essential oil from Hinoki cypress, *Chamaecyparis obtusa*, on physiology and behavior of flies. PLoS ONE.

[12] Park I.-K., Lee S.-G., Choi D.-H., Park J.-D., Ahn Y.-J. (2003). Insecticidal activities of constituents identified in the essential oil from leaves of *Chamaecyparis obtusa* against *Callosobruchus chinensis* (L.) and *Sitophilus oryzae* (L.). J. Stored Prod. Res..

[13] Hiramatsu Y., Matsui N., Ohira T., Imai Y., Miyazaki Y. (2006). Effect of hinoki (*Chamaecyparis obtusa*) wood-wool in tatami mat on the activity of house dust mite Dermatophagoides pteronyssinus. J. Wood Sci..

[14] Matsui N., Ohira T., Hiramatsu Y., Imai Y., Miyazaki Y. (2007). The composition of volatiles from tatami mats containing hinoki (*Chamaecyparis obtusa*) wood-wool and its decline over the long term. J. Wood Sci..

[15] Joo S.S., Yoo Y.-M., Ko S.-H., Choi W., Park M.-J., Kang H.Y., Choi K.-C., Choi I.-G., Jeung E.-B. (2010). Effects of essential oil from *Chamaecypris obtusa* on the development of atopic dermatitis-like skin lesions and the suppression of Th cytokines. J. Dermatol. Sci..

[16] Raha S., Kim S.M., Lee H.J., Lee S.J., Heo J.D., Venkatarame Gowda Saralamma V., Ha S.E., Kim E.H., Mun S.P., Kim G.S. (2019). Essential oil from Korean *Chamaecyparis obtusa* leaf ameliorates respiratory activity in Sprague-Dawley rats and exhibits protection from NF-κB-induced inflammation in WI38 fibroblast cells. Int. J. Mol. Med..

[17] Lee G.-S., Hong E.-J., Gwak K.-S., Park M.-J., Choi K.-C., Choi I.-G., Jang J.-W., Jeung E.-B. (2010). The essential oils of *Chamaecyparis obtusa* promote hair growth through the induction of vascular endothelial growth factor gene. Fitoterapia.

[18] Wu M.-D., Cheng M.-J., Chen J.-J., Khamthong N., Lin W.-W., Kuo Y.-H. (2022). Secondary metabolites with antimicrobial activities from *Chamaecyparis obtusa* var. formosana. Molecules.

[19] Andrews F.W. (1956). The flowering plants of the Sudan. The Flowering Plants of the Sudan.

[20] Champagnat P., Figueredo G., Chalchat J.-C., Bessière J.-M. (2006). Essential oil composition of *Vetiveria nigritana* from Mali. J. Essent. Oil Res..

[21] El Ghazali G.E.B. (1986). Medicinal Plants of Sudan, Part I: Medicinal plants of Erkowit.

[22] Ait Said L., Zahlane K., Ghalbane I., El Messoussi S., Romane A., Cavaleiro C., Salgueiro L. (2015). Chemical composition and antibacterial activity of *Lavandula coronopifolia* essential oil against antibiotic-resistant bacteria. Nat. Prod. Res..

[23] Emam M., Abdel-Haleem D.R., Salem M.M., Abdel-Hafez L.J.M., Latif R.R.A., Farag S.M., Sobeh M., El Raey M.A. (2021). Phytochemical Profiling of *lavandula coronopifolia* poir. aerial parts extract and its larvicidal, antibacterial, and antibiofilm activity against Pseudomonas aeruginosa. Molecules.

[24] Messaoud C., Chograni H., Boussaid M. (2012). Chemical composition and antioxidant activities of essential oils and methanol extracts of three wild *Lavandula* L. species. Nat. Prod. Res..

[25] Hassan W., El Gamal A., El-Sheddy E., Al-Oquil M., Farshori N. (2014). The chemical composition and antimicrobial activity of the essential oil of *Lavandula coronopifolia* growing in Saudi Arabia. J. Chem. Pharm. Res..

[26] Elsbaey M., Mwakalukwa R., Shimizu K., Miyamoto T. (2021). Pentacylic triterpenes from *Lavandula coronopifolia*: Structure related inhibitory activity on α-glucosidase. Nat. Prod. Res..

[27] Farshori N.N., Al-Sheddi E.S., Al-Oqail M.M., Hassan W.H., Al-Khedhairy A.A., Musarrat J., Siddiqui M.A. (2015). Hepatoprotective potential of *Lavandula coronopifolia* extracts against ethanol induced oxidative stress-mediated cytotoxicity in HepG2 cells. Toxicol. Ind. Health.

[28] El-Garf I., Grayer R.J., Kite G.C., Veitch N.C. (1999). Hypolaetin 8-O-glucuronide and related flavonoids from *Lavandula coronopifolia* and L. pubescens. Biochem. Syst. Ecol..

[29] El-Gendi O.D., Kusano A., KUSANO G. (2000). Two new triterpenic glucosidates from *Lavandula coronipifolia* in Egypt. Nat. Med. = 生薬學雜誌.

[30] Baptista-Silva S., Borges S., Ramos O.L., Pintado M., Sarmento B. (2020). The progress of essential oils as potential therapeutic agents: A review. J. Essent. Oil Res..

[31] Yang J., Choi W.-S., Kim J.-W., Lee S.-S., Park M.-J. (2019). Anti-inflammatory effect of essential oils extracted from wood of four coniferous tree species. J. Korean Wood Sci. Technol..

[32] Khalil M.A., Ayoub S.M.H. (2011). Analysis of the essential oil of *Vetiveria nigritana* (Benth.) Stapf root growing in Sudan. J. Med. Plants Res..

[33] Semde Z., Koudou J., Zongo C., Figueredo G., Somda M.K., Ganou L., Traore A.S. (2017). Chemical composition, antioxidant and antimicrobial activities of the essential oil of *Vetiveria nigritana* (Benth.) Stapf roots from Burkina Faso. J. Appl. Biol. Biotechnol..

[34] 34 Figueiredo A.C., Barroso J.G., Pedro L.G., Scheffer J.J.C. (2008). Factors affecting secondary metabolite produc- tion in plants: Volatile components and essential oils. Flavour Fragr. J..

[35] Yagi S., Mohammed A.B., Tzanova T., Schohn H., Abdelgadir H., Stefanucci A., Mollica A., Zengin G. (2020). Chemical profile, antiproliferative, antioxidant, and enzyme inhibition activities and docking studies of *Cymbopogon schoenanthus* (L.) Spreng. and *Cymbopogon nervatus* (Hochst.) Chiov. from Sudan. J. Food Biochem..

[36] De Almeida L.F.R., Portella R.O., Bufalo J., Marques M.O.M., Facanali R., Frei F. (2016). Non- Oxygenated sesquiterpenes in the essential oil of *Copaifera langsdorffii* Desf. increase during the day in the dry season. PLoS ONE.

[37] Alizadeh A., Aghaee Z. (2016). Essential oil constituents, phenolic content and antioxidant activity of *Lavandula stricta* Delile growing wild in southern Iran. Nat. Prod. Res..

[38] Li Y., Chen F., Li Z., Li C., Zhang Y. (2016). Identification and functional characterization of sesquiterpene synthases from *Xanthium strumarium*. Plant Cell Physiol..

[39] Sakauchi H., Kiyota H., Takigawa S., Oritani T., Kuwahara S. (2005). Enzymatic resolution and odor description of both enantiomers of lavandulol, a fragrance of lavender oil. Chem. Biodivers..

[40] El-Ahmady S.H., Ashour M.L., Wink M. (2013). Chemical composition and anti-inflammatory activity of the essential oils of *Psidium guajava* fruits and leaves. J. Essent. Oil Res..

[41] Braga P.C., Dal Sasso M., Fonti E., Culici M. (2009). Antioxidant activity of bisabolol: Inhibitory effects on chemiluminescence of human neutrophil bursts and cell-free systems. Pharmacology.

[42] Lin C.-W., Yu C.-W., Wu S.-C., Yih K.-H. (2009). DPPH Free-Radical scavenging activity, total phenolic contents and chemical composition analysis of forty-two kinds of essential oils. J. Food Drug Anal..

[43] Bouzenna H., Hfaiedh N., Giroux-Metges M.-A., Elfeki A., Talarmin H. (2017). Potential protective effects of alpha-pinene against cytotoxicity caused by aspirin in the IEC-6 cells. Biomed. Pharmacother..

[44] Karakaya S., Bingol Z., Koca M., Demirci B., Gulcin I., Baser K.H.C. (2020). Screening of non-alkaloid acetylcholinesterase and carbonic anhydrase isoenzymes inhibitors of *Leiotulus dasyanthus* (K. Koch) Pimenov & Ostr.(Apiaceae). J. Essent. Oil Res..

[45] Miyazawa M., Yamafuji C. (2005). Inhibition of acetylcholinesterase activity by bicyclic monoterpenoids. J. Agric. Food Chem..

[46] Sariri R., Seifzadeh S., Sajedi R. (2009). Anti-tyrosinase and antioxidant activity of *Lavandula* sp. extracts. Pharmacol. Online.

[47] Boutahiri S., Bouhrim M., Abidi C., Mechchate H., Alqahtani A.S., Noman O.M., Elombo F.K., Gressier B., Sahpaz S., Bnouham M. (2021). Antihyperglycemic Effect of *Lavandula pedunculata: In Vivo,* In Vitro and Ex Vivo approaches. Pharmaceutics.

[48] Ak G., Zengin G., Ceylan R., Fawzi Mahomoodally M., Jugreet S., Mollica A., Stefanucci A. (2021). Chemical composition and biological activities of essential oils from *Calendula officinalis* L. flowers and leaves. Flavour Fragr. J..

[49] Uysal S., Zengin G., Locatelli M., Bahadori M.B., Mocan A., Bellagamba G., De Luca E., Mollica A., Aktumsek A. (2017). Cytotoxic and enzyme inhibitory potential of two *Potentilla* species (P. speciosa L. and P. reptans Willd.) and their chemical composition. Front. Pharmacol..

[50] Grochowski D.M., Uysal S., Aktumsek A., Granica S., Zengin G., Ceylan R., Locatelli M., Tomczyk M. (2017). In vitro enzyme inhibitory properties, antioxidant activities, and phytochemical profile of *Potentilla thuringiaca*. Phytochem. Lett..

[51] Gerlits O., Ho K.-Y., Cheng X., Blumenthal D., Taylor P., Kovalevsky A., Radić Z. (2019). A new crystal form of human acetylcholinesterase for exploratory room-temperature crystallography studies. Chem. Biol. Interact..

[52] Rosenberry T., Brazzolotto X., Macdonald I., Wandhammer M., Trovaslet-Leroy M., Darvesh S., Nachon F. (2017). Comparison of the binding of reversible inhibitors to human butyrylcholinesterase and acetylcholinesterase: A crystallographic, kinetic and calorimetric study. Molecules.

[53] Maurus R., Begum A., Williams L.K., Fredriksen J.R., Zhang R., Withers S.G., Brayer G.D. (2008). Alternative catalytic anions differentially modulate human α-amylase activity and specificity. Biochemistry.

[54] Ielo L., Deri B., Germanò M.P., Vittorio S., Mirabile S., Gitto R., Rapisarda A., Ronsisvalle S., Floris S., Pazy Y. (2019). Exploiting the 1-(4-fluorobenzyl)piperazine fragment for the development of novel tyrosinase inhibitors as anti-melanogenic agents: Design, synthesis, structural insights and biological profile. Eur. J. Med. Chem..

[55] Karade S.S., Hill M.L., Kiappes J.L., Manne R., Aakula B., Zitzmann N., Warfield K.L., Treston A.M., Mariuzza R.A. (2021). N-Substituted valiolamine derivatives as potent inhibitors of endoplasmic reticulum α-glucosidases I and II with antiviral activity. J. Med. Chem..

[56] Omer H.A.A., Caprioli G., Abouelenein D., Mustafa A.M., Uba A.I., Ak G., Ozturk R.B., Zengin G., Yagi S. (2022). Phenolic profile, antioxidant and enzyme inhibitory activities of leaves from two *Cassia* and two *Senna* species. Molecules.

[57] Ozturk R.B., Zengin G., Sinan K.I., Montesano D., Zheleva-Dimitrova D., Gevrenova R., Uba A.I., Çakılcıoğlu U., Kaplan A., Jugreet S. (2022). Which extraction solvents and methods are more effective in terms of chemical composition and biological activity of *Alcea fasciculiflora* from Turkey?. Molecules.

[58] Morris G.M., Huey R., Lindstrom W., Sanner M.F., Belew R.K., Goodsell D.S., Olson A.J. (2009). AutoDock4 and AutoDockTools4: Automated docking with selective receptor flexibility. J. Comput. Chem..

[59] Llorent-Martínez E.J., Ruiz-Medina A., Zengin G., Ak G., Jugreet S., Mahomoodally M.F., Emre G., Orlando G., Libero M.L., Acquaviva A. (2022). New Biological and Chemical Evidences of Two *Lamiaceae* Species (*Thymbra capitata* and *Thymus sipyleus* subsp. *rosulans*): In Vitro, In Silico and Ex Vivo Approaches. Molecules.

